# Harnessing Neuroplasticity: A Case Report on Physiotherapy Rehabilitation for Millard-Gubler Syndrome

**DOI:** 10.7759/cureus.55894

**Published:** 2024-03-10

**Authors:** Anushri R Patil, Snehal Samal, Anam R Sasun

**Affiliations:** 1 Department of Neurophysiotherapy, Ravi Nair Physiotherapy College, Datta Meghe Institute of Higher Education and Research, Wardha, IND

**Keywords:** millard-gubler syndrome, case report, physiotherapy, modified ashworth scale, pontine infarct

## Abstract

This case report glances at the physiotherapy management and motor recovery outcomes of a 47-year-old female who had a pontine infarction complicated by Millard-Gubler syndrome. Pontine infarction is a stroke that occurs in the pons region of the brainstem, resulting in impaired blood flow and subsequent tissue damage. Millard-Gubler syndrome, a rare form of pontine infarction, is distinguished by ipsilateral abducens (sixth cranial nerve) and facial (seventh cranial nerve) nerve palsy, which cause horizontal gaze palsy and facial weakness, respectively. Other common symptoms include contralateral hemiparesis or hemiplegia, dysarthria, and hypertonia. In this case, the patient had nystagmus, dysarthria, hypertonia, decreased consciousness, and limited mobility. Physiotherapy interventions were used in a multidisciplinary approach to address these deficits, with a focus on improving gaze stability, reducing hypertonia, facilitating bed mobility, and improving respiratory function. The outcomes were evaluated using standardised measures such as the Brunnstrom staging for motor recovery, the Modified Ashworth Scale for hypertonia, and the Functional Independence Measure for functional status. This case demonstrates the critical role of physiotherapy in improving motor recovery and functional outcomes.

## Introduction

Pontine infarction, also known as pontine stroke, is an ischemic stroke that affects the pons region of the brain. The pons is an important part of the brainstem that regulates breathing, sleep, and communication between different parts of the brain [1]. Pontine infarctions can cause a variety of symptoms including dysphagia, impaired eye movement, and weakness in the arms and legs. There are types of pontine infarctions: Paramedian pontine infarctions are typically caused by obstructions in the basilar artery's paramedian branches, whereas tegmental pontine infarctions are caused by obstruction in the basilar artery's short circumferential branches [2,3]. Mid-pontine base infarction is caused by decreased blood flow in either the paramedian or short circumferential arteries. Multiple pontine infarctions primarily affect the territories of the perforating arteries resulting in pseudobulbar palsy due to corticobulbar fiber damage. Bilateral pontine infarctions occur when blood flow is obstructed in the larger basilar artery, affecting both sides of the brainstem [4,5]. Pontine infarctions make up 7-10% of all ischemic strokes, making them a common type of stroke. Males are more affected than females, as research on the prevalence percentage is still being continued [6]. They are prevalent in older people and are linked to a higher risk of disability and death. The ischemic insult impairs vital functions controlled by the pons, including cranial nerve function, motor control, and consciousness. Understanding the underlying mechanisms of pontine infarction is critical for tailoring treatment strategies for affected individuals [7].

Millard-Gubler syndrome is a rare condition affecting the ventral pontine area characterized by weakened eye movement (due to the abducens nerve), weakened facial muscles on the same side (facial nerve), and contralateral hemiparesis (pyramidal tract fibers) [8]. This is usually caused by a unilateral lesion at the basal portion of the caudal pons as a result of a mass, haemorrhage, or, rarely, an infarction [9,10]. Patients with pontine infarct often experience symptoms of motor deficits including weakness, spasticity, and impaired coordination. Physiotherapy interventions are focused on addressing these impairments through tailored exercise programs such as gait training and neuromuscular re-education. Furthermore, physiotherapy plays a key role in preventing secondary complications such as contractures, pressure ulcers, and respiratory complications, which can arise due to immobility and reduced muscle tone following a pontine infarct. The primary goal of physiotherapy in the case of a 47-year-old female with pontine infarction is multifaceted, with the goal of comprehensively addressing all aspects of her condition. The emphasis is on increasing consciousness through targeted interventions such as sensory and auditory stimulation. Physiotherapy helps patients regain mobility and participate in rehabilitation activities more effectively by incorporating positioning and early mobilization techniques. This includes exercises to improve coordination and encourage safe transfers to and from the bed. Furthermore, physiotherapy aims to prevent secondary complications such as pressure ulcers, contractures, and respiratory complications by using preventative measures such as skin checks and range of motion exercises. The management of spasticity is a key goal, using techniques such as stretching and positioning, therefore improving range of motion and enhancing functional abilities. Overall, the goal of physiotherapy in this case is to optimize the patient's physical function, promote recovery, and facilitate her return to a fulfilling and independent lifestyle.

## Case presentation

A 47-year-old female patient came to the hospital with complaints of loss of consciousness, fever, difficulty in breathing, and right-side hemiplegia for two months. The associated complaints include dysphagia and slurring of speech for two months. The patient was apparently alright two months ago when she suffered a sudden episode of loss of consciousness followed by a fall while standing. She was rushed to the nearest hospital by relatives, where she was admitted for eight days. The patient was shifted to the intensive care unit, where she was on a mechanical ventilator (mode was synchronized intermittent mandatory ventilation (SIMV), fraction of inspired oxygen (FiO2): 50%, and positive end-expiratory pressure (PEEP): 5 cm H_2_O); gradually on day 3, she maintained her oxygen saturation and was weaned off and was on 6 litres of oxygen support. On day 5, her oxygen support was removed. The patient was referred to physiotherapy for in-patient and out-patient rehabilitation. During the hospital stay, radiological investigations like magnetic resonance imaging (MRI) were done. Fluid-attenuated inversion recovery (FLAIR) axial MRI revealed altered signal intensity lesions in both halves of the pons and left middle cerebellar peduncle. The patient was treated with medications. Upon this, she was discharged and taken home. After two months, the patient's relative found symptoms aggravating and brought the patient to the hospital for further management. Here, an investigation like a positron emission tomography (PET) scan was done. The patient also developed septic shock, requiring the administration of ampicillin, gentamicin, and inotropic agents. The PET scan revealed heterogeneously enhancing altered signal intensity lesions in both halves of the pons and left middle cerebellar peduncle most likely suggestive of metastasis. She was referred to neurophysiotherapy for out-patient rehabilitation.

Neurological examination

After receiving oral consent, a neurological examination was performed. The vitals were hemodynamically stable. The higher functions examination revealed that the patient was conscious but not oriented to time, place, or person. The patient exhibited agitated behaviour. An additional examination revealed dysarthria and horizontal nystagmus. On motor examination, both the upper and right lower limbs had a Tone Grading Scale (TGS) score of 3+ (hypertonia), indicating an increase in muscle tone. The deep tendon reflexes for the biceps, triceps, supinator, knee, and ankle jerk were grade 3+ (exaggerated) on both sides. Babinski's sign was positive. Spasticity was assessed using the Modified Ashworth Scale (MAS) for both the upper and lower limbs; it was grade 1+ (slight increase in muscle tone, manifested as a catch, followed by minimal resistance through the remainder of the range of motion). The patient also had hypoesthesia on the right side of the body, including the face.

Diagnostic assessment

**Figure 1:**
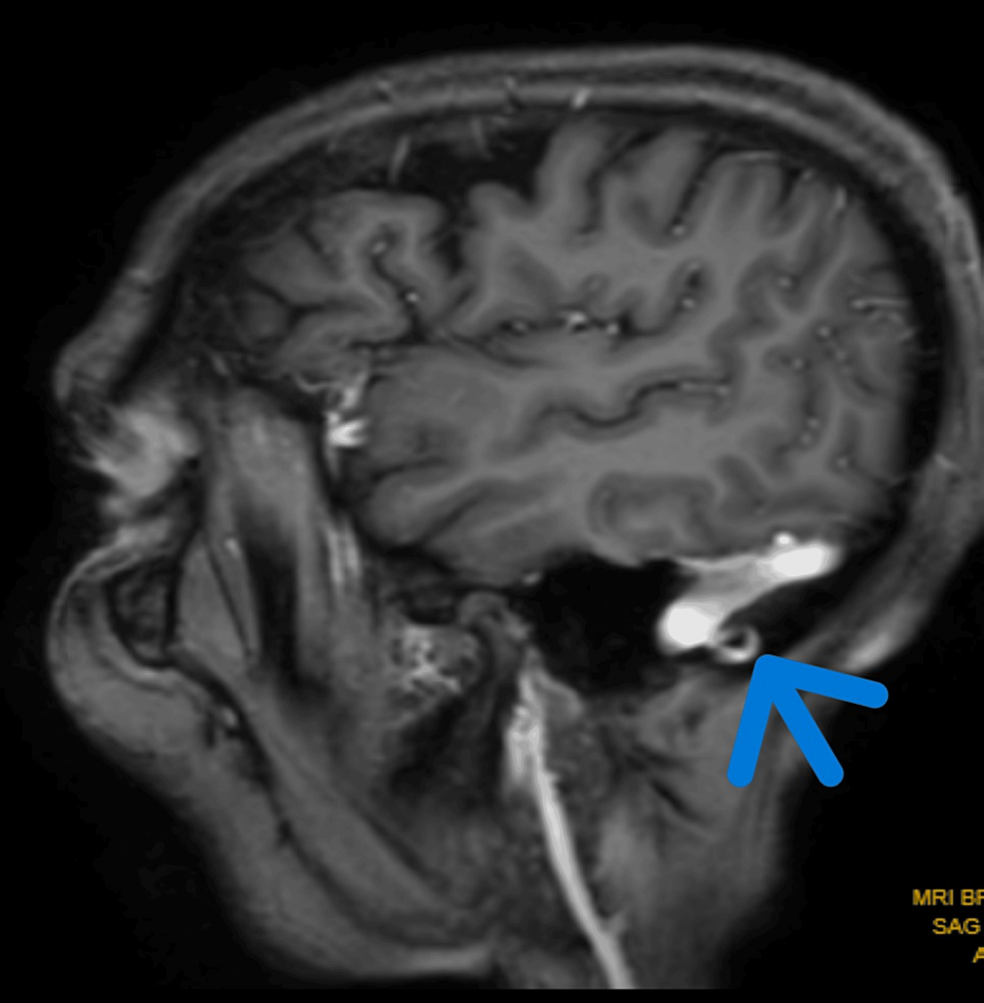
MRI of the brain, which revealed altered signal intensity lesions in both halves of the pons and left middle cerebellar peduncle MRI: magnetic resonance imaging

Figure 1 highlights the MRI of the brain, which reveals altered signal intensity lesions in both halves of the pons and left middle cerebellar peduncle.

Physiotherapy management

A six-week physiotherapy rehabilitation program was planned with the primary goal of improving patient consciousness and reducing secondary complications. Caregivers were instructed to ensure proper positioning and make constant changes every two hours. Physiotherapy rehabilitation aimed at muscle weakness, hypertonia, and bed mobility was planned, as shown in Table 1.

**Table 1:** Physiotherapy management for six weeks ROM: range of motion; PNF: proprioceptive neuromuscular facilitation; VOR: vestibulo-ocular reflex

Goals	Therapeutic exercise
Patient and caregiver education	Guidance is given to the patient's relatives about pontine infarction, its causes, and the importance of physiotherapy in preventing its secondary complication. The patient's relatives are sensitized about physiotherapy and adherence to exercise program.
To improve the level of consciousness	Multimodal stimulation including visual, auditory, and olfactory cues can be employed to engage the patient.
To inhibit hypertonia of the muscle	Rood's inhibitory techniques: inhibitory techniques involving tone regulation, like applying deep pressure on muscle tendons, gently shaking muscle bellies, compressing joints for 5-10 seconds, and maintaining prolonged stretches for 30 seconds for both upper and lower limbs (10 repetitions × 1 set).
To break synergy and aid in the activity of daily living	PNF using the rhythmic initiation technique for the upper limb and lower limb in D1 and D2 patterns, starting with passive ROM and progressing to active-assisted ROM and finally to active ROM (10 repetitions × 1 set).
To facilitate bed mobility	Initially started with supine to side lying to prone progressing to prone on the elbow side lying to sitting at the edge of the bed gradually progressing to standing beside the bed with support.
To prevent secondary complications such as bed sore, contracture, and deep vein thrombosis	Positioning every two hours with the help of pillows and educating relatives about the importance of positioning and instructing them about it. Advice water mattress instead of a regular mattress. Regular inspection of skin.
To improve symptoms of nystagmus	Gaze stabilization exercises. Smooth pursuits: keeping the head still and using the eyes to follow a moving target. Saccades: quickly move gaze between two fixed targets. VOR exercises: moving the head sideways or up and down while keeping the gaze fixed on a stationary target (10 repetitions × 1 set).
To prevent plantar flexion of bilateral ankles	The splinting technique was applied using ankle-foot orthosis.
To improve dynamic balance and posture	Sit-to-stand training with real-time visual feedback.
To improve dysarthria	Exercises like phonetic placement cues, sounds, and words in isolation were taught.
To improve cortical reorganization	Virtual reality training using Microsoft software through which games are incorporated which facilitate the use of upper and lower limbs.
To improve motor function of the bilateral upper limb	Syrebo (robotic) rehabilitation using hand gloves for grasping, gripping, pinching, and recruiting all fingers.
To improve breathing pattern	Diaphragmatic breathing exercises (10 repetitions × 1 set).
To improve hypoesthesia	Sensory organization training using various cloth textures and somatosensory discriminative training.

Follow-up and outcome measures

Table 2 features the outcome measures which were assessed pre-physiotherapy intervention and post-physiotherapy intervention.

**Table 2:** Outcome measures FIM: Functional Independence Measure

Outcome measure	Pre-physiotherapy intervention	Post-physiotherapy intervention
Brunnstrom staging [11]	Stage 3 for the upper limb and lower limb	Stage 5 for the upper limb and lower limb
FIM [12]	25	70
Stroke-Specific Quality of Life Scale [13]	49	140

## Discussion

Pontine infarctions are part of a larger ischemic event affecting the brainstem, but they can also occur only in the pons. Infarctions in this region have distinct clinical patterns resulting from cranial nerve dysfunctions and abnormalities in eye movements and involving motor skills [14]. This case report of a 47-year-old female highlights the importance of physiotherapy in overcoming the challenges and complications of pontine infarction. Physiotherapy treatment aims to improve consciousness by using auditory, visual, and tactile stimulation by means of tapping and command, etc. To reduce spasticity, inhibitory approaches were used, which were useful in improving muscle tone along with the use of proprioceptive neuromuscular facilitation (PNF) tone and improving the range of motion for the patient. Bed mobility has been an important aspect in improving the patient's tone and preventing secondary complications such as bed sore and deep vein thrombosis, which not only helped to avoid the complication but also helped to gain the patient's confidence using from supine to side lying till sitting to standing with support. Placement cues and words were incorporated to improve dysarthria, which showed significant improvement. As the patient had nystagmus, which was a problem during bed mobility, gaze stabilization exercises were incorporated which consisted of saccades and smooth pursuits with the help of objects like paper and key. Flores used balance training, mobility exercises, and gait training for a 75-year-old female following a left-sided pontine infarction and found improvement with three weeks of in-patient rehabilitation [15]. The Roods approach has been proven effective in improving hypertonia during post-stroke rehabilitation [16].

PNF has been used to reduce synergy and improve daily activities by improving tone. PNF uses the body's proprioceptive system to either facilitate or inhibit muscle contraction. Guiu-Tula et al. found its efficacy while studying stroke patients [17]. Outcome measures like the Functional Independence Measure (FIM), Brunnstrom staging of recovery, and Stroke-Specific Quality of Life Scale showed improvement after post-physiotherapy treatment [18]. Virtual reality has also been proven effective in improving mobility and cortical recognition in stroke patients. It gives a new dimension to enhance the quality of life [19,20]. Physiotherapy plays a crucial role in improving the overall health and mobility of patients with pontine infarcts, along with active instructions for the family members of patients [20].

## Conclusions

Physiotherapeutic rehabilitation for Millard-Gubler syndrome has proven to be an important part of our 48-year-old patient's comprehensive management. We have seen significant improvements in consciousness, motor function, and overall quality of life after following a tailored regimen of exercises, manual therapy, and targeted interventions. The importance of physiotherapy cannot be overstated, as it not only addresses physical impairments but also promotes neuroplasticity and recovery. This case emphasises the importance of a multidisciplinary approach, with physiotherapy playing a critical role in improving functional outcomes and the well-being of Millard-Gubler syndrome patients.
